# Supplementary material for: Melatonin-Induced Inhibition of Shiraia Hypocrellin A Biosynthesis Is Mediated by Hydrogen Peroxide and Nitric Oxide
Source: J Fungi (Basel). 2022 Aug 10;8(8):836. doi: 10.3390/jof8080836 (PMC9410495; doi:10.3390/jof8080836)
Supplement: Supplementary file 1 [file jof-08-00836-s001.zip › jof-1840657-supplementary.pdf]

Electronic supplementary material

Melatonin-induced Inhibition of *Shiraia* hypocrellin A Biosynthesis Is Mediated by Hydrogen Peroxide and Nitric Oxide

Wen Juan Wang [1], Qun Yan Huang [1], Yue Wang[1], Xin Ping Li [1], Jian Wen Wang [1], Li Ping Zheng[2,*]

*Correspondence: lpzheng@suda.edu.cn; Tel.: +86-521-65880195

Table S1. Primer and relevant information of reference and target genes.
F: forward primer, R: reverse primer.

Genes	Gene Description	Name of Primer	Sequence
18S	Reference gene	*18S*-F	5'-ACGCAGCGAAATGCGATAAG-3'
		18S-R	5'-CAAATTGTGCTGCGCTCCAA-3'
ZFTF	zinc finger transcription factor	*ZFTF*-F	5'-GAACACCGTCGCAAGATTCG-3'
		ZFTF-R	5'-TCATTGGCATCGCTTGGAGT-3'
PKS	Polyketide synthase	*PKS*-F	5'-GCAGATACGCCCCTCACTAC-3'
		PKS-R	5'-GTCGCTGGTAATATCGCCCA-3'
Omef	*O*-methyltransferase	*Omef*-F	5'-GGGAGAGCGATACGCATTCA-3'
		Omef-R	5'-TACTGCAGCTGTCACCAAGG-3'
FAD	FAD/FMN-containing dehydrogenase	*FAD*-F	5'-ACGAGGTTTGGCATCGTCAT-3'
		FAD-R	5'-ACGAATATGCCCGACTCCAC-3'
Mono	Salicylate 1-monooxygenase	*Mono*-F	5'-TCGCCCGAACTCTATCAAGC-3'
		Mono-R	5'-ACTCTCCTGAAAACCGAGCG-3'
MCO	Multicopper oxidase	*MCO*-F	5'-TATGGCGCTACGAGTGGAC-3'
		MCO-R	5'-ACTCCCTGGCCGATAACGTA-3'
MFS	Major facilitator superfamily	*MFS*-F	5'-CAGAAAGCAAGGCTACGGGA-3'
		MFS-R	5'-TCACCAAATCGCCGAAGGAA-3'
